# Spontaneous Deletion of an “ORFanage” Region Facilitates Host Adaptation in a “Photosynthetic” Cyanophage

**DOI:** 10.1371/journal.pone.0132642

**Published:** 2015-07-15

**Authors:** Richard J. Puxty, Blanca Perez-Sepulveda, Branko Rihtman, David J. Evans, Andrew D. Millard, David J. Scanlan

**Affiliations:** 1 School of Life Sciences, University of Warwick, Coventry, West Midlands, CV4 7AL, United Kingdom; 2 Warwick Medical School, University of Warwick, Coventry, West Midlands, CV4 7AL, United Kingdom; University of Strathclyde, UNITED KINGDOM

## Abstract

Viruses have been suggested to be the largest source of genetic diversity on Earth. Genome sequencing and metagenomic surveys reveal that novel genes with unknown functions are abundant in viral genomes. Yet few observations exist for the processes and frequency by which these genes are gained and lost. The surface waters of marine environments are dominated by marine picocyanobacteria and their co-existing viruses (cyanophages). Recent genome sequencing of cyanophages has revealed a vast array of genes that have been acquired from their cyanobacterial hosts. Here, we re-sequenced the cyanophage S-PM2 genome after 10 years of near continuous passage through its marine *Synechococcus* host. During this time a spontaneous mutant (S-PM2d) lacking 13% of the S-PM2 ORFs became dominant in the cyanophage population. These ORFs are found at one loci and are not homologous to any proteins in any other sequenced organism (ORFans). We demonstrate a fitness cost to S-PM2^WT^ associated with possession of these ORFs under standard laboratory growth. Metagenomic surveys reveal these ORFs are present in various aquatic environments, are likely of cyanophage origin and appear to be enriched in environments from the extremes of salinity (freshwater and hypersaline). We posit that these ORFs contribute to the flexible gene content of cyanophages and offer a distinct fitness advantage in freshwater and hypersaline environments.

## Introduction

Bacteriophages are the most numerous biological entities on Earth [1]. Through cell lysis they impact biodiversity [2] and biogeochemical cycles [3]. Moreover, bacteriophages alter the evolutionary trajectories of host organisms through transduction and through antagonistic co-evolution [4–6]. Amongst the best studied are those phages infecting the environmentally important marine picocyanobacteria (cyanophages). Marine picocyanobacteria comprise two main genera *Synechococcus* and *Prochlorococcus*, that together contribute ~25% of global CO_2_ fixation [7], and are the most numerous photoautotrophs on Earth. Over a decade of genome sequencing of cyanophage isolates has revealed diverse hypothetical phage-host interactions including augmentation of photosynthesis [8–10], carbon metabolism [11] and phosphate acquisition [12]. However, the lack of genetic systems in these phages has prevented gaining an understanding of the role of cyanophage hypothetical proteins in the infection process. These proteins are responsible for around 30% of the cyanophage specific gene content and therefore contribute significantly to the vast genetic diversity observed in these viruses [13]. Moreover, whilst a core-set of cyanophage specific genes can be identified [14–16], the contribution of the flexible genome to niche adaptation is yet to be rigorously established. At the extremes of this flexible gene set lie the ORFans: ORFs lacking any orthologues in other sequenced genomes.

During evolution of bacteria to a fixed environment, rate discordance is observed, whereby despite a near linear increase in genomic substitution, phenotypic fitness benefits are only rapidly evolved during early generations [17]. The evolution of bacteriophages to a fixed host is expected to follow such observations, with one important caveat; the genome of their host represents a rich source of exogenous genetic material that, through recombination, may provide novel gene combinations, allowing for the rapid development of new phenotypes. Indeed, the sequencing of bacteriophage genomes points to diverse host-derived gene acquisitions [14,15,18], which have been suggested to play a role in metabolic re-programming *in infecto* to support phage growth [11,19–22]. To understand the rates and diversity of gene gains and losses and indeed to detect possible occurrences of horizontal gene transfer (HGT), we re-sequenced the cyanophage S-PM2 after 10 years of near constant passage through its host, the marine cyanobacterium *Synechococcus* sp. WH7803.

**Fig 1:**
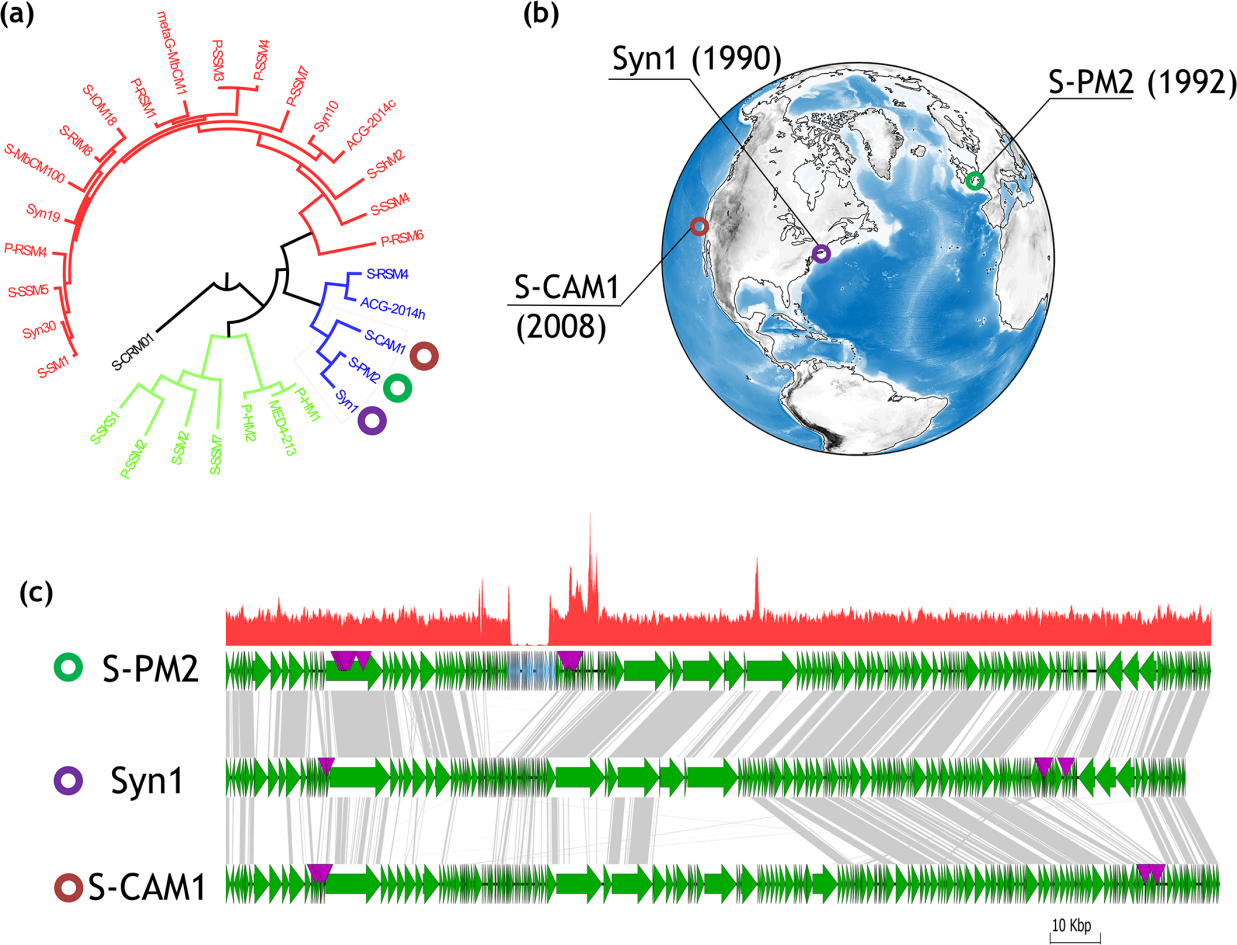
Phylogenetic analysis, isolation sites and ORFan presence in S-PM2. (a) Phylogenetic reconstruction of the sequenced T4-like cyanophages showing the sub-cluster containing S-PM2, Syn1 and S-CAM1. Phylogenetic reconstruction is based on the amino acid alignment of gp20 sequences. (b) Map showing the year and site of isolation of cyanophages S-PM2, Syn1 and S-CAM1. (c) Genetic map of S-PM2 and closely related cyanophages Syn1 and S-CAM1. Orthologous ORFs are linked by grey lines. Locations of tRNAs are shown by purple triangles. Blue filled ORFs in the S-PM2 panel show the locations of the ORFans. Top panel shows reads mapped to S-PM2 from high-throughput sequencing.

S-PM2 is an obligately lytic myovirus with a genome ~196 kb in size, encoding 244 ORFs [23]. Comparative genomics with other sequenced marine cyanomyoviruses suggests that S-PM2 is an “outlier” [15]. This is due to the fact that S-PM2 lacks many of the marine cyanophage “nearly-core” genes identified in [15]. Of particular interest is the paucity of so called “auxiliary metabolic genes” (AMGs *sensu* [24]) in S-PM2, in comparison with other marine cyanophages [15]. Despite this, S-PM2 still possesses a relatively large genome, containing genes that are unique amongst other cyanophages. Indeed, a contiguous region of the genome from ORF 017 to 050 contains 33 ORFans and as such has been called an “ORFanage” region [25]. This ORFanage region can be clearly distinguished when compared with the closely related cyanophages Syn1 and S-CAM1 (Fig 1A and 1C), which were isolated from disparate locations over an 18 year period (Fig 1B).

Here, we show that during continuous passage, the aforementioned “ORFanage” region was deleted from S-PM2^WT^. The loss of these ORFs is concomitant with an increase in fitness under laboratory conditions as detected by an increase in the rate of plaque growth. We show that these ORFs are frequently found in metagenomic datasets from diverse environments and seem to be enriched in those of extreme salinity, both freshwater and hypersaline environments. The ORFs are frequently found in the same neighbourhood yet the synteny observed in S-PM2^WT^ is rarely conserved. This indicates intense “shuffling” of genes at this locus. The possible mechanisms and consequences of this shuffling are discussed in relation to the evolution of this group of viruses.

## Materials and Methods

### Growth of strains and phages

*Synechococcus* sp. WH7803 was grown is ASW medium [26] at 23˚C in continuous light at 10–30 μmol photons m^-2^ s^-1^. Cyanophage S-PM2d was prepared from lysates by aspiration of top agar, polyethelene glycol (PEG) precipitation and CsCl gradients as described in [14,23].

### Cyanophage S-PM2d DNA extraction and sequencing

Genomic DNA was extracted from S-PM2d lysate. Cell debris was discarded by centrifuging at 3220 g for 20 min at 4°C and the supernatant used for DNA extraction using phenol/chloroform and precipitated with NaAc/ethanol [23]. Proteinase K and SDS were added to a final concentration of 20 mg ml^-1^ and 0.5% (w/v) respectively, and incubated at 60°C for 4 hr. An equal volume of phenol was added to the sample followed by centrifugation for 5 min at 16060 g. The aqueous layer was then mixed with an equal volume of chloroform:isoamyl alcohol (24:1 v/v) and centrifuged for 5 min at 16060 g. 0.1 volumes of 3.5 M sodium acetate were added to the aqueous layer. The sample was incubated overnight at -20°C with excess ethanol and centrifuged at 16060 g for 30 min at 4°C. The supernatant was discarded and the pellet washed with 1 mL 70% (v/v) ethanol. The sample was incubated for 15 min at 4°C and centrifuged at 16060 g for 20 min at 4°C. The pellet was re-suspended in 100 μL nuclease free water (Ambion) and purified with DNeasy Plant mini spin columns (Qiagen, Vinlo, Netherlands) and quantified by NanoDrop and QuantiFluor dsDNA System (Promega, Madison, USA). Illumina library preparations were performed at the Centre for Genomic Research, University of Liverpool, using the TruSeq protocol (Illumina, San Diego, U.S.A) with 1 μg of DNA. Libraries were sequenced with the MiSeq platform generating 2 x 250 bp reads.

### Read alignment and mutation detection

Raw sequences were trimmed for Illumina adaptors using Cutadapt v1.1 [27] option-O 3 and further trimmed using Sickle v1.2 [28] with a minimum window quality score of 20 by the Centre for Genomic Research, University of Liverpool. Reads were aligned to the S-PM2^WT^ genome (Acc. No. NC_006820.1) using Bowtie v2 [29] with the “very-sensitive” option. In total, 62,634 reads aligned concordantly exactly once yielding a mean coverage of 152.9x±28.1.

Samtools v0.1.18 [30] was used for SAM file manipulation. For conversion to binary format Samtools view options “-bS-F4” were used, with the mpileup file produced using the S-PM2^WT^ genome (Acc. No. NC_006820.1) as a reference, and with BAQ disabled.

Mutations were detected using VarScan v2.3.3 [31] option—p-value 0.01,—min-coverage 8,—min-avg-qual 30,—min-var-freq 0.9 (pileup2snp for SNPs and pileup2indel for Indels). All mutations were checked manually.

### Genome assembly

Trimmed reads (see above for details) were used for cyanophage S-PM2d genome assembly using SPAdes 3.1 [32]. Annotations from S-PM2^WT^ were transferred onto S-PM2d. The complete assembled S-PM2d genome sequence was deposited in EMBL-EBI under the accession number LN828717.

### Confirmation of deletion

The large deletion that was identified through genome sequencing was confirmed by PCR. Three primer pairs were designed that detected the deleted ORFs (Del1_F/R = 5’- GTTTCCCCGATGACTTACGA-3’ / 5’- GGACATTCCCAGTCCTCAAA-3’, Del2_F/R = 5’- GGTGCTTGATGCTCGTGATA-3’ / 5’- GGACAGCATCCCATTTTTGT-3’, Del3_F/R = 5’- GCTGACCTTGCTGCTAATCC-3’ / 5’- GAATTGGGTCCACACGTTCT-3’) and also 4 primer sets that flanked the deletion site (Flank1_F/R = 5’-GAGAACTCCTGGTGGAGCTG-3’ / 5’-TCTGCTGGAGAGCATCACAC-3’, Flank2_F/R = 5’-GAGAACTCCTGGTGGAGCTG-3’/ 5’-AATGCTGTCACGACGATCAC-3’ Flank3_F/R = 5’-CACTCGTGCTAAAGCTGCTG-3’/ 5’-TCTGCTGGAGAGCATCACAC-3’ Flank4_F/R = 5’-CACTCGTGCTAAAGCTGCTG-3’ /5’-AATGCTGTCACGACGATCAC-3’). PCR reactions were performed in a 50 μl volume, containing 1x MyTaq master mix (BioLine, London, UK), 0.4 μM primers and water to 50 μl. Cycling conditions were 60 s at 95°C followed by 35 cycles of denaturation at 95°C for 15 s, annealing at 55˚C for 15 s and extension at 72°C for 30 s. Template was ~45 ng of DNA from lysates of S-PM2 from 2006 or 2013. These lysates were shown to contain mixed populations of S-PM2^WT^ and S-PM2d. Therefore, we isolated individual plaques from these mixed populations and carried out PCR to determine possession of the ORFanage region. PCR reactions on isolated plaques were accomplished by aspiration of the soft agar containing the plaque into 100 μl ASW medium in each well of a 96 well plate and 1 μl was subsequently used as template for the PCR. PCR products (10 μl) were separated on a 1% (w/v) agarose gel and visualised with ethidium bromide.

### Determination of the fitness cost of cyanophage S-PM2d using plaque assays

To determine the fitness cost associated with the S-PM2 deletion, the rate of plaque growth was measured as described in [26]. In short, increasing dilutions of phage stocks were plated with the *Synechococcus* WH7803 host and incubated under constant illumination of 10–30 μmol photons m^-2^ s^-1^ until plaque appearance. For plaque size comparison, measurements were taken from plates which had plaques sufficiently distant from one another to ensure measurement of single plaques. Thirty four plaques from each of the phage strains were randomly picked from plates of different dilutions. Plaque size was determined by analysing images of plates using ImageJ [33] To convert the pixel size to radius, the images were taken next to a standard ruler and a distance of 1 mm used as a standard and was measured to be equal to 25 pixels.

### Metagenomic analyses

The deleted S-PM2 ORFs were searched for in publically available metagenomic datasets from CAMERA (http://camera.calit2.net/) downloaded 21/01/2014. The 33 deleted ORFs were first searched against the CAMERA databases using tblastn with the following parameter settings:-evalue 10^−5^, word_size 3, gapopen 11, gapextend 1. Hits were filtered based on 40% nucleotide identity to the query and 50% coverage. The fact that many of the ORFs were “ORFans” was exploited by using best reciprocal blast hit (BRBH) against the NCBI-nr database. Only hits whose BRBH was the corresponding ORF from S-PM2 were included for further analysis. Relative gene abundance (RGA) was calculated as:

$$RGA=\frac{\left(N/Q_{L}\right)}{{DB}_{s}}x10^{12}$$

where N is the number of positive hits, Q_L_ is the query length and DB_S_ is the size of the database size in nucleotides.

Metagenomic reads containing a positive hit were further searched against the NCBI–nr database using tblastn with the following parameter settings:-evalue 10^−5^, word_size 3, gapopen 11, gapextend 1 to discover potential ORFs co-localised to the same fragment. Again, a threshold of 40% nucleotide identity to the query and 50% coverage was used to filter hits.

Moreover, some metagenomic fragments were sequenced in a paired end fashion. Therefore corresponding mate reads of reads containing positive hits were recruited from the database. To attempt to detect the organismal origin of the read, the corresponding pair was used in a tblastn search using the parameter settings:-evalue 10^−5^, word_size 3, gapopen 11, gapextend 1 of the NCBI–nr database.

### Phylogenetic analysis

To identify cyanophage genomes that are closely related to S-PM2 we reconstructed a phylogeny from the gene encoding the portal vertex protein, *gp20*. Amino acid sequences of gp20 from 31 sequences cyanophages were downloaded from NCBI (Accession numbers: YP_004323020.1, YP_007877943.1, YP_004324725.1, YP_004323264.1, YP_004323950.1, YP_009007965.1, YP_007518198.1, YP_008126421.1, YP_007877738.1, YP_007001618.1, YP_008129949.1, YP_214665.1, YP_004324951.1, ACD93441.1, YP_007001830.1, YP_004322786.1, YP_007677272.1, YP_007675137.1, YP_003097343.1, YP_009008243.1, YP_007673103.1, YP_195138.1, YP_004324491.1, YP_004322541.1, YP_007673752.1, YP_004323487.1, YP_004324197.1, YP_004322270.1, YP_214363.1, YP_007674507.1, YP_004508471.1). Alignments were performed using muscle with the following parameter settings: gapopen -2.9, gap extend 0 and manually refined. Phylogeny was inferred using maximum likelihood methods using the WAG+I+Γ_4_ substitution model as implemented in MEGA v5.2.

## Results and Discussion

To identify any mutations in the re-sequenced S-PM2 genome (S-PM2d) that may have been selected for during propagation of this cyanophage over ~10 years, a SNP and indel analysis was carried out in comparison to the S-PM2^WT^ genome. A total of 3 SNPs, 4 insertions and 7 deletions were identified by whole genome sequencing (S1 Table) and confirmed by PCR and Sanger sequencing. Since some of these mutations resulted in restoration, elongation or fusion of ORFs that were consistent with potential orthologues from other cyanophages (S1 Table), we hypothesised that these may be the result of errors in the original S-PM2 genome sequence (Accession No. NC_006820.1). To this end, we used PCR and Sanger sequencing to detect these mutations in S-PM2 lysates from as far back as 1994. These mutations were indeed present in previous lysates and thus all of the apparent mutations were the result of original erroneous genome sequencing. These mutations have been corrected in the deposited EMBL-EBI file of S-PM2d (Accession No. LN828717).

**Fig 2:**
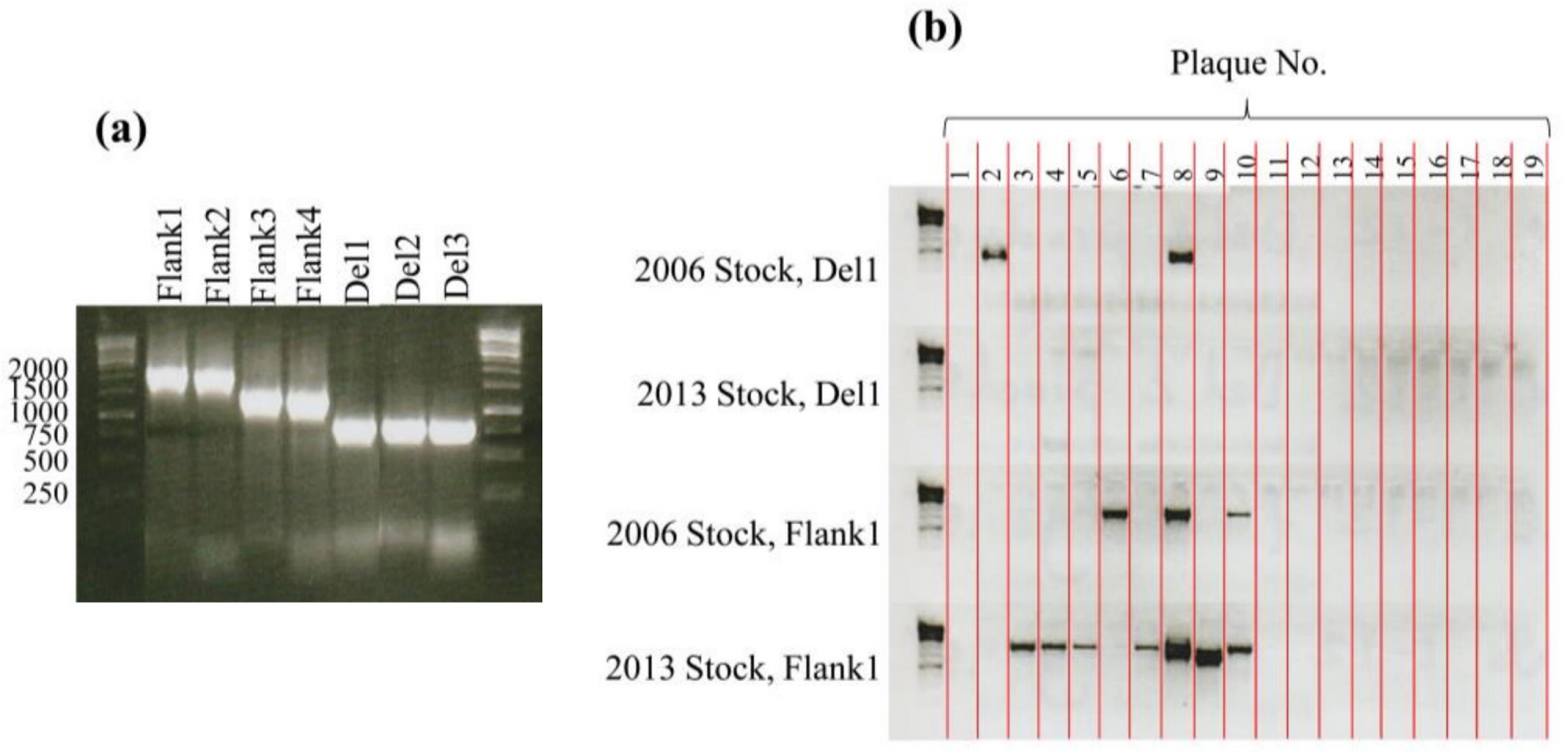
Detection of the ORFanage deletion by PCR. (a) PCRs using a crude S-PM2 lysate from 2013 as template. ‘Flank1, 2, 3, 4’ primer sets are designed to flank the ORFanage region and should yield an amplicon of ~10 kb each. ‘Del 1, 2, 3’ primer sets are designed to target sites internal to the ORFanage region. Lanes 1 and 9 represent the 1kb ladder (Fermentas, Life Technologies, Carlsbad, U.S.A.). (b) Flank1 and Del1 primers were used to target 19 isolated plaques from S-PM2 lysates from 2013 and 2006. Absence of a band indicates the PCR was unsuccessful.

However, no reads were mapped to a ~10 kb region of the S-PM2 genome (Fig 1C), suggesting that this region may have been deleted. To validate this, PCR was used with primers internal to (‘Del’) and flanking the potential deletion (‘Flank’) (Fig 2). This showed that the deleted ORFs were present in recent lysates of cyanophage S-PM2 from 2013 (Fig 2A, lanes 6, 7, 8). Interestingly, PCR analysis also showed that products were obtained for the targets ‘Flank1-4’. This was surprising given that these amplicons should be greater than 10 kb in length. These amplicons were estimated to be ~1.25–2 kb by gel electrophoresis (Fig 2A, lanes 1–4). Thus, it was clear that a deletion had occurred in a fraction of the population of cyanophage S-PM2 virions from lysates after 2013. Sequencing of the Flank1 amplicon revealed that the deletion had occurred between genomic coordinates 5,799 and 15,339 of the original cyanophage S-PM2 sequence. This was further confirmed by assembly of the reads into a single contiguous genome. Thus, 9,540 nucleotides had been deleted from the genome encompassing 33 ORFs from S-PM2p017 to 050. This represents 4.9% of the genome and 13.5% of the S-PM2^WT^ ORFs. Therefore, two cyanophage S-PM2 variants existed that herein are referred to as S-PM2^WT^ and S-PM2d. To estimate the frequency of each variant, PCR was carried out on 19 isolated plaques from S-PM2 lysates produced in 2013 and 2006. 2/3 of the PCR positive clones from 2006 were cyanophage S-PM2d (Fig 2B). One plaque (8) contained products from both Flank1 and Del1 PCRs and is probably indicative of a mixed plaque. In comparison, no plaques of cyanophage S-PM2^WT^ were obtained from the 2013 stock, whereas 7 were obtained that were cyanophage S-PM2d. Taken together with the absence of reads that mapped to the deleted region indicates that the frequency of S-PM2^WT^ is extremely low in lysates of cyanophage S-PM2 from 2013.

Deletions of this size are rarely reported in the literature. What is more interesting is that these ORFs form an apparent “ORFanage” region [23,25] (Fig 1). Indeed, orthologues of the deleted ORFs cannot be found in the NCBI-nr database, whilst those ORFs surrounding the deletion are readily found in cyanophage genomes (Fig 1).

**Fig 3:**
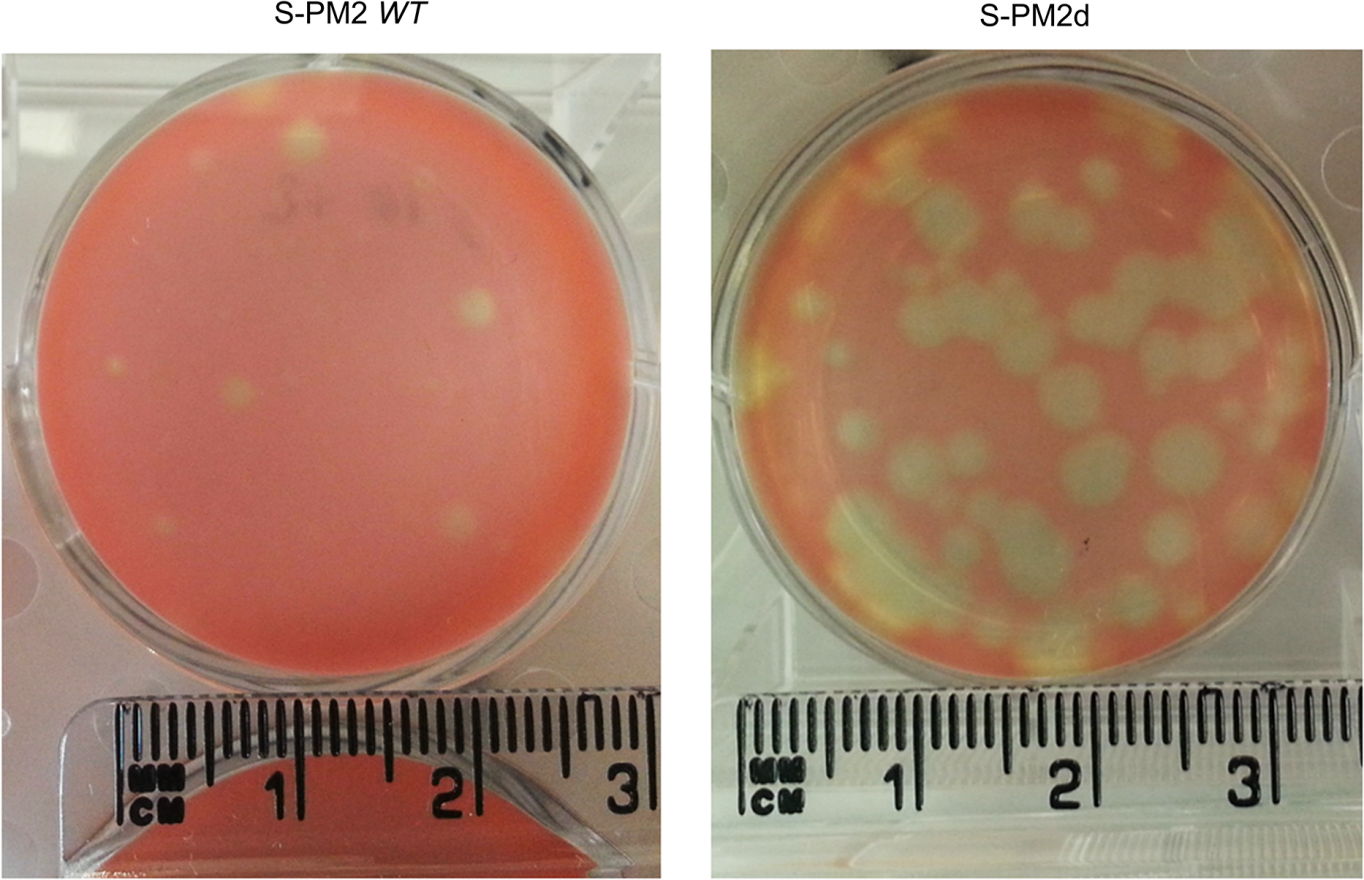
Representative plaques of S-PM2^WT^ and S-PM2^d^.

For S-PM2d to become the dominant variant during routine passage, we hypothesised that there must be a fitness cost associated with maintenance of these ORFs. To test this, plaque growth was monitored between isolated plaques of S-PM2^WT^ and S-PM2d on lawns of *Synechococcus* sp. WH7803 (Fig 3). Plaques of S-PM2^WT^ were consistently significantly smaller than those of S-PM2d by approximately 6-fold (t (34) = 8.6445 *p*<0.0001). This is indicative of slower rates of viral growth [34] in S-PM2^WT^ and thus there appears to be a reduction in fitness with maintenance of the ORFs under standard laboratory conditions. The reduction in fitness conferred by these ORFs raises an important facet to the nature of cyanophage gene gain and loss. Is negative selection for deleterious genes particularly weak or do these ORFans offer positive selection in certain environmental niches? The former implies an inherent level of stochasticity that may make the understanding of viral gene gain and loss rather complex, whilst the latter suggests that the flexible cyanophage protein space may contribute to niche specialisation. To begin to tease apart these effects, we sought to understand the abundance of genes orthologous to these ORFans in metagenomic datasets. In so doing we hypothesised that their abundance may be correlated with some environmental variable.

**Fig 4:**
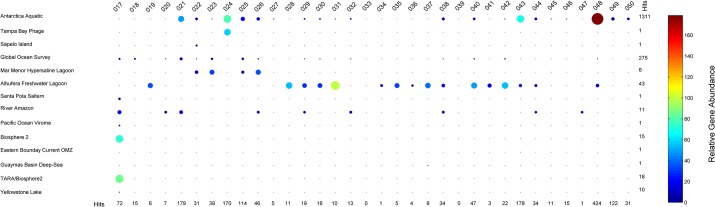
Relative abundance of the deleted ORFans in metagenomic datasets. ORFs are described by the ORF number relative to S-PM2. CAMERA accessions for metagenomic datasets are as follows: TARA/Biosphere 2 (CAM_P_00001027), Guaymas Deep Sea Basin (CAM_P_0000545), Eastern Boundary Current OMZ (CAM_P_0000692), Biosphere 2 (CAM_P_0000912), Pacific Ocean Virome (CAM_P_0000914), River Amazon (CAM_P_0001128), Santa Pola Saltern (CAM_P_0001130), Albufera Freshwater Lagoon (CAM_P_0001132), Mar Menor Hypersaline lagoon (CAM_P_0001133). The relative abundance is shown by the colour bar and is proportional to the size of the circle.

**Fig 5:**
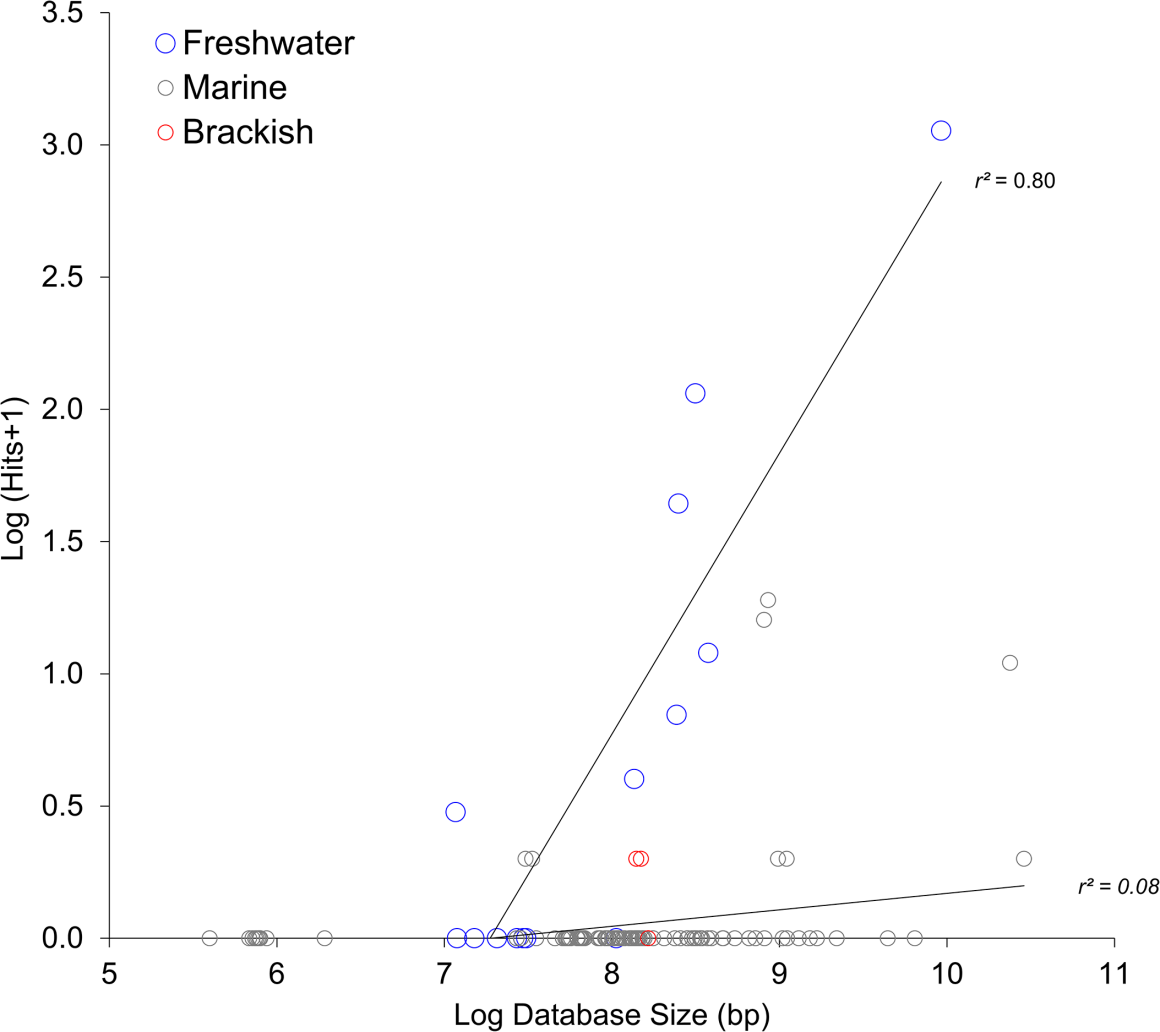
Selection for ORFans in non-marine aquatic environments. Correlations between database size and frequency of deleted ORFs in metagenomic samples classified as marine, freshwater or brackish.

Thirty six metagenomic datasets were screened for potential orthologues (see materials and methods). After filtering, 1695 hits were found on 1602 discrete reads from 14 libraries searched (Fig 4; S2 Table). The normalised abundance of these orthologues in each of the 14 metagenomes containing hits are shown in Fig 4. Orthologues appeared to be enriched in metagenomes from the extremes of salinity (i.e. freshwater or hypersaline environments). In particular, orthologues were abundant in Yellowstone Lake, Mar Menor hypersaline lagoon, Albufera freshwater lagoon and River Amazon metagenomes. Orthologues were also found in the Global Ocean Survey (GOS) metagenome. However, a closer inspection revealed that almost all were from freshwater or hypersaline environments (stations GS012, GS020, GS033), with the exception of two hits coming from stations GS013 and GS014 which are coastal sites of intermediate salinity [35]. With this in mind, metagenomes were categorised by their salinity contained within the available metadata. The database size and number of hits were strongly positively correlated with freshwater sites (Pearson’s moment correlation, *r* ^*2*^(14) = 0.80, *p*<0.001, Fig 5). A positive correlation also exists between the database size and the number of hits for marine sites, but the correlation is far weaker (*r* ^*2*^(111) = 0.08, *p* = 0.003, Fig 5). A closer inspection of those marine sites that contained positive hits reveal that 3 contain only hits to S-PM2 ORF 017, which, whilst partially deleted in S-PM2d, is not an ORFan, sharing similarity with hypothetical genes from a wide range of bacteria (data not shown). Removal of these sites from the analysis results in a non-significant correlation between database size and number of hits for marine sites (*r* ^*2*^(111) = 0.16, *p* = 0.09).

**Fig 6:**
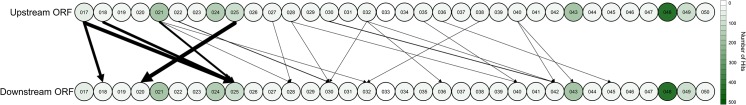
Synteny plot of deleted ORFs from S-PM2 co-localised on metagenomic fragments. The width of the arrow is proportional to the frequency of the association between the two ORFs. Circles are coloured according to the total number of hits of each ORF in all metagenomic datasets.

Moreover, there is differential abundance of the ORFs within each metagenome, suggesting that these ORFs are not part of some conserved genetic module. To test this we searched for contiguous deleted ORFs on the same metagenomic fragment. The majority (215/218) of reads containing contiguous hits came from the GOS database, owing to large read lengths. The other three reads came from the Amazon River metagenome (1) and the Yellowstone Lake metagenome (2). Fig 6 shows that the S-PM2 deleted ORFs can routinely be found in the same gene neighbourhood. However, the synteny and polarity are rarely conserved. A much more common arrangement is for S-PM2p017 or S-PM2p018 to be succeeded by S-PM2p025. Moreover, a frequent observation is for S-PM2p025 to be found upstream of S-PM2p020. ORFs at the 3′ end of the deleted region of S-PM2 are rarely found on the same metagenomic fragment, this is despite high abundances of ORFs S-PM2p043 and S-PM2p048. Therefore, it is likely that these ORFs originate from disparate loci in other genomes and have since converged into this region of S-PM2. Taken together, these data point to this region being a hotspot for gene exchange.

Lastly, we sought to identify the origin of potential orthologues from metagenomic datasets. Therefore, metagenomic fragments containing hits to the deleted ORFs were subjected to BLAST analysis against the NCBI-nr database. Of the 1602 reads we could only identify 6 (0.004%) that contain another ORF whose best BLAST hit (BBH) was not of cyanophage origin (S2 Table), showing highest similarity to uncultured phages (1), *Polynucleobacter necessaries* (3), *Herpetosiphon aurantiacus* (1) and *Clostridium botulinum* (1). All of these reads came from the GOS dataset and therefore we sought the corresponding mate pair read to give greater confidence to the taxonomic association. Of these mates, 4 contained ORFs whose BBH was similar to a cyanophage ORF and are therefore of likely cyanophage origin. One mate contained no ORFs of similarity to anything in the–nr database, whilst the final mate contained two ORFs showing similarity to both a *Synechococcus* phage and *Herpetosiphon aurantiacus*. Moreover, we recruited all mate pair reads that contained hits to deleted ORFs and carried out BLAST analysis against the NCBI–nr database. We identified 9 (0.006%) mate pair reads that contained a non-cyanophage BBH. The BBHs contained on these reads were from a *Salmonella* phage (1), *alpha proteobacterium SCGC AAA015-O19* (1), *Polynucleobacter necessaries* (2), *Synechococcus sp*. WH7803 (1), *Herpetosiphon aurantiacus* (1), *Clostridium botulinum* (2) and *Calothrix* sp. PCC 6303 (1). Of these 9 reads, 5 contained at least 2 ORFs on the corresponding mate pair whose BBHs were cyanophages and thus are likely of cyanophage origin.

In summary, we isolated and identified a deletion mutant of S-PM2 that is lacking the complete ORFanage region as previously described [23,25]. Maintenance of the ORFanage region confers a fitness cost to S-PM2^WT^. Metagenomic analysis suggests positive selection of these ORFs in freshwater and hypersaline environments and that we could not conclusively identify any of these ORFs in a non-cyanophage like organism. Therefore, we hypothesise that these ORFs may offer some niche adaptation to cyanophage lytic growth under extremes of osmotic gradients. Very few sequenced isolates of cyanophages from freshwater or hypersaline environments exist. Dreher et al., (2011) sequenced the freshwater cyanophage S-CRM01 and show that 186 (63%) ORFs are ORFans. This data, together with the findings of this paper suggest that the freshwater cyanophage protein space is vastly undersampled. Moreover, given that S-PM2 was isolated from a coastal site, a significant amount of horizontal gene transfer may exist between these environmental reservoirs.

The mechanism for such a recombination event between S-PM2 or its progenitor and a hypothetical freshwater phage remains unclear. Recently, a role for promoter early stem loops (PeSLs) has been demonstrated for mediating shuffling of genes between closely related T4-like phages [36,37]. Interestingly, we can identify a host-like early promoter upstream of *g016* (5322–5350) [23] and potential stem loops after S-PM2p*038* (11369–11391) and S-PM2p*055* (16829–16854). However, this is distinct from the case in other T4- like phages where a much higher density of PeSLs are observed in regions of intense shuffling [36,37]. Therefore, it is unlikely that this mechanism is responsible for acquisition of these ORFs. This is especially true in light of the synteny observed on metagenomic fragments. Here, it appears that the synteny of these ORFs is rarely conserved, suggesting they are not part of a mobile module and instead are the result of independent acquisitions. We note the proximity of the ORFanage region to a region of increased read density (~2–3 fold) immediately downstream of the ORFanage region (Fig 1C). We interpret this signature as resulting from terminal redundancy of phage DNA ends [38]. T4 phage have circularly permuted and terminally redundant ends [39]. That is, DNA concatamers are cut by the T4 terminase at variable loci at least 1 headful package length away from the *pac* sites [40–43] and approximately 3% extra DNA is packaged into the head yielding terminally redundant ends. Thus, for each copy of the genome, approximately 2 copies exist downstream of the cut sites compared with upstream, resulting in increased read density. We therefore propose that the ORFanage region was lost from S-PM2 by a mispackaging event resulting in packaging of a truncated genome into the phage head. Similarly, foreign genetic material may be acquired at this locus by illegitimate recombination during genome concatemerisation. Thus, this may represent a novel mechanism by which horizontally acquired genes can be “tested”. DNA conferring a negative fitness cost can thus be rapidly lost from the genome by inaccurate packaging events. Further evidence to support this hypothesis may be gained by examining termini of phage DNA and their relationship to the genomic positions of ORFans.

## Supporting Information

S1 Table Corrections to the original S-PM2 genome sequence. (XLSX)

S2 Table Results of BLAST searches of metagenomic datasets for ORFan genes. (XLSX)
